# Operationalizing ambiguity in sustainability science: embracing the elephant in the room

**DOI:** 10.1007/s11625-023-01446-6

**Published:** 2023-12-15

**Authors:** Anita Lazurko, L. Jamila Haider, Tilman Hertz, Simon West, Daniel D. P. McCarthy

**Affiliations:** 1 School of Environment, Resources, and Sustainability, University of Waterloo, 200 University Avenue W, N2L 3G1 Waterloo, Canada; 2 UK Centre for Ecology and Hydrology, Library Avenue, Bailrigg, Lancaster, LA1 4AP UK; 3 Stockholm Resilience Centre, Stockholm University, Stockholm, Sweden; 4 Crawford School of Public Policy, Australian National University, Canberra, Australia; 5 Northern Institute, Charles Darwin University, Darwin, Australia

**Keywords:** Transdisciplinarity, Ambiguity, Boundaries, Complexity, Co-production

## Abstract

Ambiguity is often recognized as an intrinsic aspect of addressing complex sustainability challenges. Nevertheless, in the practice of transdisciplinary sustainability research, ambiguity is often an ‘elephant in the room’ to be either side-stepped or reduced rather than explicitly mobilized in pursuit of solutions. These responses threaten the salience and legitimacy of sustainability science by masking the pluralism of real-world sustainability challenges and how research renders certain frames visible and invisible. Critical systems thinking (CST) emerged from the efforts of operational researchers to address theoretical and practical aspects of ambiguity. By adapting key concepts, frameworks, and lessons from CST literature and case studies, this paper aims to establish (1) an expansive conceptualization of ambiguity and (2) recommendations for operationalizing ambiguity as a valuable means of addressing sustainability challenges. We conceptualize ambiguity as *an emergent feature of the simultaneous and interacting boundary processes associated with being, knowing, and intervening in complex systems*, and propose Reflexive Boundary Critique (RBC) as a novel framework to help navigate these boundary processes. Our characterization of ambiguity acknowledges the boundary of a researcher’s subjective orientation and its influence on how ambiguity is exposed and mediated in research (being), characterizes knowledge as produced through the process of making boundary judgments, generating a partial, contextual, and provisional frame (knowing), and situates a researcher as part of the complexity they seek to understand, rendering any boundary process as a form of intervention that reinforces or marginalizes certain frames and, in turn, influences action (intervening). Our recommendations for sustainability scientists to operationalize ambiguity include (1) nurturing the reflexive capacities of transdisciplinary researchers to navigate persistent ambiguity (e.g., using our proposed framework of RBC), and (2) grappling with the potential for and consequences of theoretical incommensurability and discordant pluralism. Our findings can help sustainability scientists give shape to and embrace ambiguity as a fundamental part of rigorous sustainability science.

## Introduction

Transformative change is required for humanity to overcome the root causes of twenty-first-century environmental crises (United Nations 2015; Patterson et al. 2017). There is growing agreement that for sustainability science to contribute to this change, it must bridge the science-society interface through action-oriented and integrative knowledge production (Cornell et al. 2013; Fazey et al. 2020; Caniglia et al. 2020). The paradigms of transdisciplinarity and knowledge co-production have emerged in response to this call, offering promising contributions to the future of sustainability science (Lang et al. 2012; Brandt et al. 2013; Klenk and Meehan 2017; Chambers et al. 2022). Yet, in their effort to make sense of and influence contemporary sustainability issues, these paradigms grapple with the persistent ambiguity, broadly understood as the potential for multiple valid frames, that is an inherent feature of complex sustainability challenges (Preiser et al. 2018; Dewulf et al. 2020). This ambiguity surfaces through engagement with the plural values and perspectives of diverse actors involved in knowledge production and persists through knowledge production that resists integration via any singular disciplinary frame (Leach et al. 2010; Turnhout et al. 2020).

The ambiguity that permeates transdisciplinary research creates several challenges. Ambiguity generates misunderstanding when collaborating across paradigms (Strang 2009; Turnhout 2019), and potentially incommensurate frames may emerge from diverse theory orientations informed by different ontological (ways of being) and epistemological (ways of knowing) commitments (Kuhn 1970; Hertz and Schlüter 2015). In a common metaphor (i.e., the ‘blind observers and the elephant’), researchers and other participants in co-production processes are standing too close to—or blindly grasping for—part of the elephant (i.e., reality) to embrace the partial, and ambiguous, contributions their observations play in relation to a complex whole. Amid the need for action- and solutions-oriented sustainability research (Miller et al. 2014; Clark et al. 2016), these paradigmatic differences have real-world consequences. For example, Hertz and Mancilla Garcia (2021) demonstrate that in the case of the 1980s Baltic Sea cod fisheries collapse, the underlying worldview informing the science, policy, and practice of cod fishing (i.e., of humans as separate from nature) contributed to overfishing, which may have been mitigated by a shift toward a relational worldview (i.e., humans and nature as fundamentally intertwined). Additionally, local and Indigenous knowledges are increasingly called upon for their unique contribution to more holistic understandings of environmental change (Klenk and Meehan 2015; Rathwell et al. 2015). However, knowledge integration processes can be risky as these marginalized frames may be co-opted, reduced, or instrumentalized by more dominant scientific perspectives (Ocholla 2007; Stein et al. 2020; Goodchild 2021). Moreover, in the cases of disagreement or incommensurability between frames (e.g., between a critical social science and natural science perspective), more dominant frames are viewed as neutral and objective while marginalized frames are cast as political or subjective (Turnhout 2018; Turnhout et al. 2020). For example, the development of a monitoring and evaluation (M&E) framework for natural resource management in Australia aimed to avoid the risks of cooption or instrumentalization of Indigenous frames by centering Indigenous methodologies. This process enhanced the framework’s legitimacy in the eyes of Indigenous rangers, while paradoxically reducing its comprehensibility (and thus potentially its legitimacy) for some non-Indigenous scientists, planners, and policy actors (Campion et al. 2023). Such real-world implications of ambiguity are discussed by Brugnach and Ingram (2012), who posit that failures of more integrative natural resource management can be attributed to a mishandling of ambiguity.

While the challenges of ambiguity emerge through research practice, ambiguity itself is a slippery concept. The literature on uncertainty first recognized ambiguity in differing interpretations of numbers (Funtowicz and Ravetz 1990) and the subjectivity of a model’s system boundaries (Walker et al. 2003). Dewulf and Biesbroek (2018) broadened this definition, defining ambiguity as “conflicts between fundamentally different frames about the issue at hand” and differentiating ambiguity as distinct from epistemic uncertainty (i.e., lack of knowledge) and ontological uncertainty (i.e., inherent variability). In sustainability science, ambiguity has been discussed through the dominant social-ecological systems (SES) perspective, which views linked human-natural systems as complex adaptive systems (Folke 2016; Reyers et al. 2018). From this view, “we cannot know complex things completely” (Cilliers 2002), so ambiguity arises from complexity because any knowledge excludes pertinent system components and relationships (Matthews 2006; Preiser et al. 2018, 2021). Further, any claims to be holistic about complexity can be considered political, as any expertise depends on choices that define a system and how it can be improved (Sarewitz 2010). Science and technology studies also highlight ambiguity as political and emergent from pluralism, where actors produce divergent framings that interact and challenge dominant system structures (Leach et al. 2010; Stirling 2014). This interpretation reveals how ambiguity involves the inextricability of epistemology and ontology—i.e., framings are interventions that both emerge from and shape future action. Thus, ambiguity appears to be a feature of complexity, emerging from the intersection of the subjectivity and partiality of knowledge and its impact on the systems in which this knowledge is produced. However, the precise shape and origins of ambiguity, including its onto-epistemological dimensions, remain unclear. Referring to the ‘blind researchers and the elephant’ metaphor: why do researchers see, smell, hear, or feel a different part of the elephant from others? Or are they seeing different animals entirely?

Different literature operationalizes aspects of the challenges presented by ambiguity. For example, TD researchers are embracing the multiple frames produced by diverse knowledge systems through limited forms of integration, weaving together multiple frames to develop an enriched picture while maintaining their individual integrity (e.g., Martin 2012; Tengö et al. 2014). Miller et al. (2008) propose a reorganization of academic research to enable such epistemological pluralism, with a particular focus on bridging across disciplinary or paradigmatic frames (Kuhn 1970). The STEPS pathways to sustainability approach also grapples with epistemological pluralism via the interaction between constructivist perspectives (which produce critical reflection between different framings) and positivist perspectives (which present a single objective reality), and how they can together inform more holistic and pluralist sustainability research (Leach et al. 2007). Most of this literature focuses on the epistemological domains of ambiguity, but sustainability researchers are also drawing from seminal work that addresses the multiple frames produced by *ontological* pluralism, thereby completely avoiding integration (Goodman 1978; Escobar 2018). For example, Vervoort et al. (2015) suggest Goodman’s ‘worldmaking’ as an appropriate framework for imaginative transdisciplinary processes that aim to contribute to sustainability transformation, because it enables ‘ontological agency’ (i.e., by building out futures as independent worlds rather than different narratives of the same world). Emerging literature also takes a more reflexive perspective on ambiguity by turning attention back on the researchers themselves to explore the role of a researcher’s positionality and capacity to navigate the ambiguities of transdisciplinary research (e.g., Haider et al. 2018; Chambers et al. 2022).

This existing literature conceptualizes and operationalizes aspects of ambiguity, offering hints of how it can be understood and addressed in sustainability science. Yet, much of sustainability science still operates from a middle space, neither situated comfortably within a singular frame nor explicitly aware of or addressing the elephant in the room—i.e., ambiguity. Sustainability scientists operating in this middle space may embrace complexity and understand the need for pluralism broadly but struggle to overcome their tendency to evaluate knowledge against a singular ‘unambiguous’ frame. In such cases, ambiguity is not explicit yet persists, leaving research vulnerable to the risks and power dynamics associated with uncritical knowledge integration and transdisciplinary collaboration. Sustainability science needs new concepts and tools to operationalize ambiguity in an expansive and reflexive way, which can further strengthen the legitimacy of transdisciplinarity as a research paradigm and make the adaptive and emergent nature of the transdisciplinary research journey more explicit and deliberate (McGowan et al. 2014). Moreover, doing so can aid sustainability scientists trying to enter—or gesture toward—the ‘ethical space’ between frames required for truly pluralist transdisciplinary research (Goodchild 2021).

Operational research has a multi-decade history grappling with ambiguity, offering an opportunity for sustainability science. Operational research began with the use of hard systems models underpinned by expert-driven positivism, followed by a second wave of soft systems approaches underpinned by an interpretivist perspective (Midgley 1989; Flood and Jackson 1991; Jackson 2019). Divergence and conflict between first and second-wave approaches emerged alongside the observation that understandings of a problem and what constitutes ‘improvement’ may change significantly when system boundaries are altered (Churchman 1970). Thus, Churchman’s pragmatist critique of the systems approach launched a third critical-emancipatory wave called critical systems thinking (CST) underpinned by tenets of critical awareness, emancipation, and pluralism (Flood and Ulrich 1990; Gao et al. 2003; Matthews 2006). CST explicitly grappled with both the conceptual challenges associated with ambiguity, including theoretical/methodological pluralism and paradigm incommensurability (Midgley 1989, 1992; Ulrich 2003), and the need for practical frameworks that operationalize ambiguity through reflection on system boundaries (Ulrich 1983; Midgley 2000).

Emerging research points to the promising lens offered by CST for sustainability research (e.g., Helfgott 2018; Rutting et al. 2022), yet the use of CST concepts and tools is still marginal. Thus, we were motivated by the opportunity to bridge key concepts, frameworks, and lessons from CST literature to the challenges presented by ambiguity in sustainability science. The resulting insights aim to (1) establish an expansive conceptualization of ambiguity that addresses its onto-epistemological dimensions while prioritizing its operationalization, and (2) offer recommendations for how sustainability scientists can operationalize our conceptualization of ambiguity as a valuable means of addressing sustainability challenges. We first introduce our rationale for using system boundaries as the primary lens from which to conceptualize and operationalize ambiguity in sustainability science. We then introduce and explain our conceptualization of ambiguity before offering two overarching recommendations for sustainability scientists to operationalize ambiguity.

## The importance of system boundaries

Ambiguity, understood broadly as the potential for multiple valid frames, is often discussed as a feature of complex systems. Complexity emerged from the systems approach and has been studied from various perspectives (Bateson 1979; Prigogine and Stengers 1984; Rosen 1991; Cilliers 1998; Levin 1999). Following conflicts between the first and second waves of systems thinking (Midgley 1992, 2011), critical systems theorists emphasized the role of pragmatism in operational research, which views all knowledge as partial, contextual, and contingent, as it is “impossible to apprehend (non-contextually) the whole system” (Churchman 1970; Matthews 2006). This view drew attention to the importance of system boundaries in defining the limits of any particular frame of a system. These boundaries (and the resulting frames) are not value-free and fixed entities determined by the structure of reality but rather depend on the subjective and value-laden choices of individuals setting and reinforcing them. A recent epistemological break moved away from the restricted complexity of this systems approach (i.e., studying specific types of systems called “complex”) toward general complexity (i.e., the view that any system is complex), drawing attention to the relationship between the whole system and its parts (Morin 2008; Preiser et al. 2018). From this view, boundaries can be understood as that which frame yet also constitute the system (Cilliers 2002). This view highlights the pervasive and persistent nature of ambiguity, where multiple valid frames are not only possible, but the entities that produce a frame and its validity are also part of the system and thus delineated by boundaries. CST gives these boundaries and the resulting ambiguity some language and shape by focusing attention on the sources of selectivity in the system, i.e., the process of making boundary judgments related to motivation, power, knowledge, or legitimacy, which generates a dominant view of which facts or values are relevant (Ulrich 1983).

The view of ambiguity as emerging from boundary processes is compatible with the dominant SES perspective in sustainability science, which views linked human and natural systems as complex adaptive systems (CASs). CASs are characterized by unique features, such as dynamic relations and complex causality (Levin et al. 2013; Preiser et al. 2018). These features further explain the contextual, partial, and provisional nature of boundaries. For example, CASs are radically open as information, energy, and matter are constantly exchanged across a permeable boundary between the system and its environment (Preiser et al. 2018, 2021). They are also constituted relationally, meaning a system’s behavior is determined more by the nature of its interactions than individual components, and these interactions connect systems in nested hierarchies across spatial and temporal scales (Gunderson and Holling 2003; Cash et al. 2006; Preiser et al. 2018). These features render the external boundary conditions as integral to system behavior as the system structure, and make it nearly impossible to decide which system components are inside and outside the system (Juarrero 1999; Preiser et al. 2018). Thus, any representation of the system is dependent on subjective boundaries. These chosen boundaries generate one of multiple partial frames that includes certain components and exclude others. Further, they are dependent on the choices of the observer who is also a part of the system they seek to understand (Cilliers 2001; Audouin et al. 2013; Preiser et al. 2018).

## Conceptualizing ambiguity in sustainability science

### An expansive and operational conceptualization of ambiguity

**Fig. 1:**
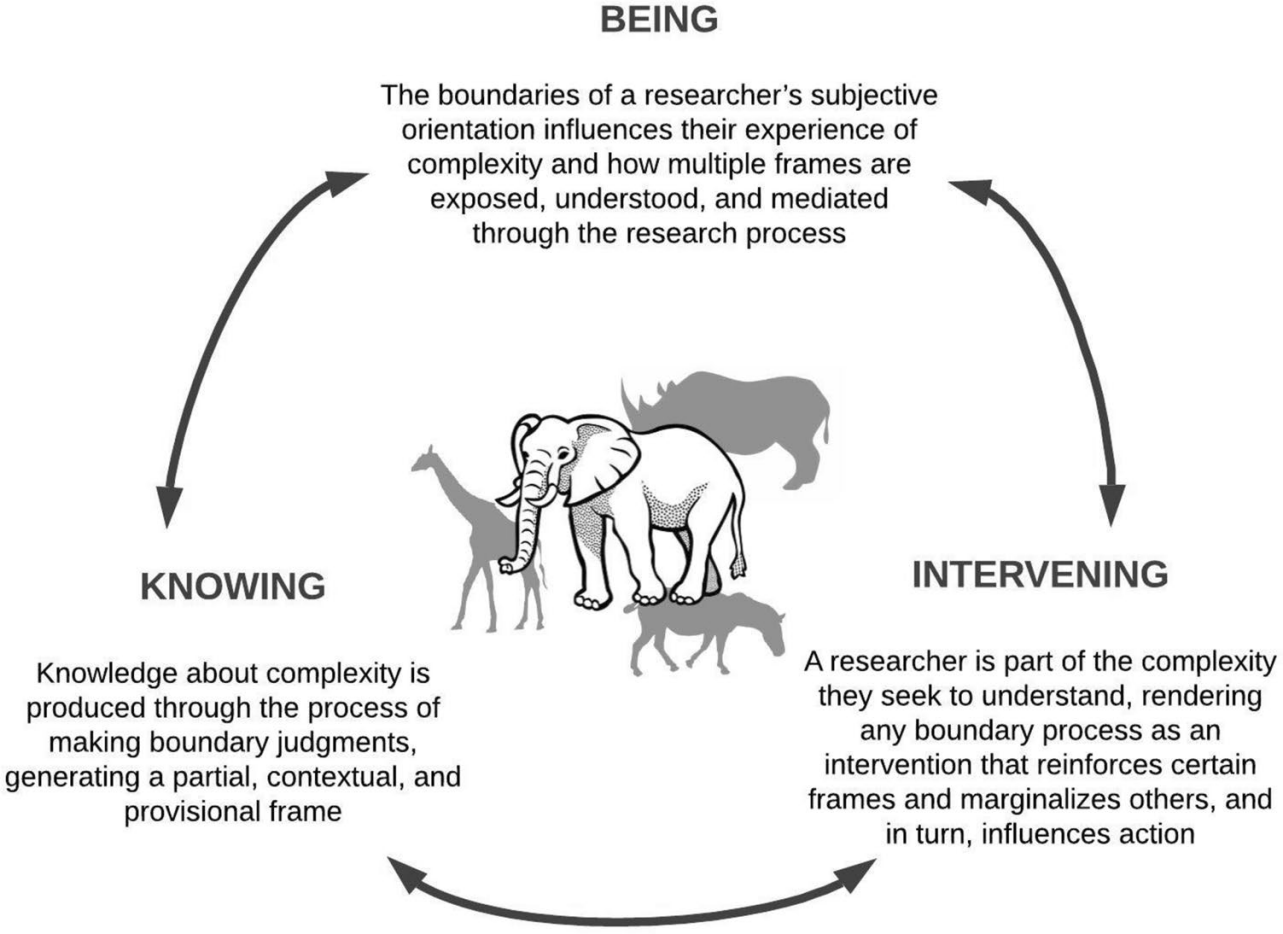
The visual depiction of an operational definition of ambiguity—an emergent feature of the simultaneous and interacting boundary processes of being, knowing, and intervening in complex systems. This conceptualisation of ambiguity modifies the ‘blind observers and the elephant’ metaphor described in the introduction. Rather than different actors seeing different parts of the elephant, our understanding of ambiguity suggests that actors might be seeing different animals altogether

Given the importance of system boundaries described in "The importance of system boundaries", we offer a conceptualization of ambiguity focused on boundary processes. We define ambiguity as an *emergent feature of the simultaneous and interacting boundary processes associated with being, knowing, and intervening in complex systems*. This definition draws on three considerations as depicted in Fig. 1. First, "Processes of being: observer-dependence" (observer dependence) demonstrates that an operational definition of ambiguity must acknowledge the boundaries of a researcher’s subjective orientation. These boundaries influence their experience of complexity and how multiple frames are exposed, understood, and mediated through the research process (*Being*). Second, "Processes of knowing: knowledge as a boundary process" (knowledge as a boundary process) demonstrates how knowledge about complexity is produced through the process of making boundary judgments, generating a partial, contextual, and provisional frame (*Knowing*). This frame may be one of multiple valid frames of a complex system. Third, "Processes of intervening: boundary marginalization" (boundaries as intervention) demonstrates how a researcher is part of the complexity they seek to understand, rendering any boundary process as an intervention that reinforces certain frames and marginalizes others, and in turn, influences action (*Intervening)*. These three processes interact with one another in complex ways, producing emergent ambiguity.

Our conceptualisation of ambiguity departs from conventional definitions (e.g., as the potential for multiple valid frames) in important ways. First, we do not offer a concrete definition of exactly what ambiguity is, but rather describe ambiguity as an emergent feature of complexity, always in the process of becoming. This conceptualisation deliberately avoids concretizing ambiguity as something that can be completely understood and resolved, but rather asks of us to navigate it as an ever-present and dynamic part of sustainability science. Second, we view ambiguity as emergent from diverse epistemological *and ontological* positions, which highlights how multiple frames can be equally valid but mutually exclusive. In other words, each frame is distinct and may only be intelligible from a particular ‘horizon of intelligibility’, i.e., a set of individual material-discursive practices (Schatzki 2002) or situated position (Haraway 1988). This conceptualisation leaves open the possibility for discordant frames that generate a deep and persistent form of ambiguity that further defies resolution. Finally, our definition of ambiguity centers the political and ethical nature of any knowledge claims about complexity by highlighting how research involves subjective choices that render particular frames visible or invisible.

### Processes of being: observer-dependence

Our definition of ambiguity acknowledges the boundaries of a researcher’s subjective orientation, which influences their experience of complexity and how multiple frames are exposed, understood, and mediated through the research process (i.e., *processes of being*). This contribution emerged through reflection on the lessons of CST literature regarding the observer-dependence of system boundaries. Here, observer-dependence refers to the individual subjectivities that define the boundaries of a researcher’s subjective orientation. Such subjectivities are discussed in literature across the social sciences. For example, critical and feminist theories emphasize how knowledge is socially situated and thus implicates personal positionality related to gender, age, race, or geographic context (Haraway 1988; Harding 1995). Such literature is relevant to understanding the observer-dependence of sustainability science (e.g., Rosendahl et al. 2015; Chambers et al. 2022; Staffa et al. 2022). CST complements this view by focusing on how theoretical and methodological pluralism contributes to ambiguity by generating multiple—and potentially incommensurate—frames among researchers, which in turn affects the boundary processes that occur within the research process.

Theoretical and methodological pluralism is an epistemological principle for CST, which describes and organizes the simultaneous use and integration of various systems approaches adopted by researchers, which produce diverse frames of a system. This principle emerged from attempts to reconcile debates between first- and second-wave system approaches by recognizing that methodologies derived from different or contradictory paradigms (e.g., positivist versus interpretivist frames) offer different sources of selectivity that generate valid but partial and contextual framings of a system (i.e., boundaries). While desirable in theory, “atheoretical pragmatism” surfaced in practice as individuals picked and chose methodologies without knowledge of their theoretical origins (Midgley 1992; Bowers 2019). This was perceived as a threat to the field, because the subjectivities of the individual systemists could become a primary source of selectivity in the choice of theory or method, rather than a more coherent set of rules. As a result, critical system theorists sought an appropriate meta-theoretical framework to guide systemists who were operationalizing pluralism in practice (Bowers 2011). Such a framework was meant to minimize the influence of an individual’s subjective frame (i.e., observer dependence) in selecting a theory or method, thereby improving the legitimacy of the boundary processes that follow. The system of systems methodology (SOSM) is the most prominent of such attempts, guiding which type of methodologies are appropriate for the type of system (i.e., simple, complex) and the relationship between participants (i.e., unitary, pluralist, coercive) (Jackson and Keys 1984; Jackson 2019).

While useful for orienting the field of CST, the use of meta-frameworks like SOSM was criticized for two reasons. First, they were too rigid, as *how* a methodology is used is as important as the theoretical context (Bowers 2011; Jackson 2019). For example, the same systems approach could produce different frames of the same system if different stakeholders were included in its application. Thus, some focus turned to the capacities of individual systemists as they navigated within and across theories and methodologies through boundary choices, influenced by the limits of their subjective frames and their broader context (Bowers 2019). Second, these meta-frameworks were considered problematic as any meta-theoretical framework is itself a frame, which is still delineated by subjective boundaries. Thus, the assumption that a meta-theory exists at all assumes theoretical commensurability, wherein all frames must in some sense ‘agree’ to fit within the boundaries of a larger meta-frame (Gregory 1996). This assumption of theoretical commensurability may appear to reconcile the ambiguity that emerges from the diverse theoretical and methodological frames, thereby establishing legitimacy despite the subjectivity (i.e., observer dependence) required to navigate such pluralism. However, this assumption emphasizes pluralism as complementarism focused on consensus and integration, which risks masking incommensurate frames (i.e., those that do not ‘agree’) through an (inadvertent) form of imperialist pluralism (Gregory 1996; Ulrich 2003; Jackson 2019). In other words, an assumption of theoretical commensurability can mask ambiguity, as discordant frames are discarded or rendered invisible through the process of integration with other frames. In response, some critical systems theorists encouraged *discordant* pluralism, which embraces the observer dependence of boundary processes by assuming that any claims about a system are contingent, local, and historically situated, and promotes communication between radically different perspectives (Gregory 1996).

A conceptualisation of ambiguity that directly addresses observer dependence through the lens of theoretical and methodological pluralism is highly relevant to sustainability science. Transdisciplinary researchers operate from diverse disciplinary and theory orientations that produce different frames of sustainability challenges (Miller et al. 2008; Hertz and Schlüter 2015). For example, ‘resilience thinking’ (Folke et al. 2010; Folke 2016) and the ‘pathways to sustainability approach’ (Leach et al. 2010) are prominent paradigms in sustainability science. Both apply a systems approach but are rooted in divergent ontological and epistemological origins and thus call on different theories and methods, producing different frames of a system and recommendations for action (West et al. 2014; Haider et al. 2018). However, despite widespread acceptance of the benefits of such theoretical pluralism, the dominance of certain theory orientations (i.e., the SES perspective) can lead researchers to adopt their own as a meta-framework that describes reality, rather than offering one partial and contingent frame (West et al. 2020). In such cases, the boundaries of the researcher’s frame are rendered invisible, as frames that are incommensurate with the SES perspective (e.g., due to differing onto-epistemological origins) are instrumentalized or discarded as they are subsumed under its purview. Such concerns deepen the risks of ‘epistemological sovereignty’ discussed by Miller et al. (2008) with consideration of ontological pluralism, which highlights the possibilities for incommensurability and discordance between frames. Relatedly, best practice frameworks for integrating knowledge system s (e.g., scientific, Indigenous, and local knowledge) aim to allow each to maintain the integrity of its frame (Tengö et al. 2014; Hill et al. 2020). However, these frameworks are not mainstream, and epistemological and ontological differences between frames can generate potentially discordant perspectives that challenge the integration imperative of sustainability science (Klenk and Meehan 2015; Turnhout 2019; Cockburn 2022).

### Processes of knowing: knowledge as a boundary process

Our definition of ambiguity characterizes knowledge about complexity as produced through the process of making boundary judgments, generating a partial, contextual, and provisional frame (i.e.,* processes of knowing*)*.* This contribution emerged through reflection on CST’s principle of boundary critique and lens of process philosophy and their relevance to the system ontology of dominant perspectives in sustainability science.

CST deals with observer dependence ("Processes of being: observer-dependence") by focusing attention on the subjective boundary judgments that are required during knowledge production to generate the boundaries of any frame of system. According to Churchman, boundaries are social and personal constructs that determine the limits of knowledge that are considered pertinent for an analysis (Churchman 1970). Critical Systems Heuristics (CSH) was proposed as a framework to guide boundary critique, i.e., critical reflection upon how subjective boundaries are generated and their consequences (Ulrich 1983; Ulrich and Reynolds 2010). According to CSH, any claim about a system depends on a reference system, which is made up of *boundary judgments* that generate the dominant view of which facts and values are relevant (Ulrich 1983). These boundary judgments are understood as any sources of empirical or normative selectivity that influence the frame of a system, extending beyond choices typically regarded as ‘boundary choices’ (e.g., spatial scale) to include broad sources of motivation, power, knowledge, and legitimacy. For example, the measure for desirable change in a system may be a source of motivation, who has a stake in the system may be a source of power, and whose knowledge ‘counts’ in the system may be a source of knowledge or legitimacy. CSH includes a list of questions designed to facilitate boundary critique by revealing these sources of boundary judgments, and questions are asked in both the ‘is’ mode and the ‘ought’ mode to reveal contested and unresolved judgments (Jackson 2019). CSH thus reveals how any frame of a system emerges from a series of implicit and explicit boundary judgments.

Critical systems theorists iterated on the framework of CSH to establish a broader philosophy of knowledge focused on boundary processes. In particular, Midgley’s (2000) process philosophy is a philosophy of knowledge for CST that views knowledge as emergent from continuously unfolding boundary processes. Midgley’s process philosophy was inspired by the process philosophy pioneered by Whitehead (1978). It proposes a shift from the *content* of knowledge (i.e., what we know) to the *process* of bringing knowledge into being (i.e., how we come to know it), in particular the *process of making boundary judgments*. Midgley (2000) claims this form of process philosophy is more suitable for the use of systems approaches to inform intervention than other philosophies of knowledge. This claim is based on its deviation from the assumption held by other theories, i.e., that independent observation is possible through the separation of the observer and the observed (e.g., Popper’s critical fallibilism, Kelly’s personal construct theory, and Habermas’ Three Worlds). Instead, Midgley’s process philosophy views both the observed (i.e., boundaries of the system) and the observer (i.e., observer dependence; what it is that gives rise to these boundaries) through the same lens (i.e., the process of making boundary judgments). In doing so, Midgley’s process philosophy situates a systemist as within the system, which is compatible with a complexity perspective that situates observers as part of the complexity they seek to understand. Moreover, it centers the observer dependence of knowledge in the analysis, facilitating theoretical and methodological pluralism by allowing different frames emerging from different boundary processes reflecting diverse theoretical or methodological orientations to co-exist without contradiction (Midgley 2000; Jackson 2019).

A conceptualisation of ambiguity that gives shape to the boundary processes that produce multiple valid frames is currently missing in sustainability science. Discussion on boundaries in particular has focused on using ambiguous concepts as boundary objects, wherein the potential for multiple interpretations of the concept (e.g., resilience, stewardship) serves as a tool to facilitate dialog among different perspectives (Brand and Jax 2007; Peçanha Enqvist et al. 2018). Similarly, boundary work is the discursive process that delineates science from non-science in complex sustainability issues (Gieryn 1983; Miller 2013). Perhaps most relevant is the work of Audouin et al. (2013), who draw on critical complexity (Preiser and Cilliers 2010) to suggest five key questions that can surface the value judgments behind any framing of an SES. However, none of these domains address the processes by which multiple frames are produced, nor deeper onto-epistemological considerations that implicate individuals and their subjectivities in these processes (i.e., observer dependence). Interestingly, the emerging ‘process-relational turn’ of sustainability science advocates for a greater focus on *processes and relations* in research, as a counterpoint to the more mainstream substantialist view that focuses on *structures and objects* (Hertz et al. 2020; West et al. 2020). The former tends to critique the latter for necessitating concrete and strict boundaries that render certain frames and solutions invisible. Thus, a conceptualization of ambiguity informed by Midgley’s process philosophy (i.e., focused on boundary processes) not only speaks directly to the system ontology of more established domains (e.g., the SES perspective), but may serve as a bridge to this emerging strand of sustainability science (i.e., process-relationality).

### Processes of intervening: boundary marginalization

Finally, our definition of ambiguity situates a researcher as part of the complexity they seek to understand, rendering any boundary process as an intervention that reinforces certain frames and marginalizes others (i.e.,* processes of intervening*). This statement emerged through reflection on key boundary-related frameworks from CST and their relevance to the political and ethical implications of ambiguity in sustainability science.

**Fig. 2:**
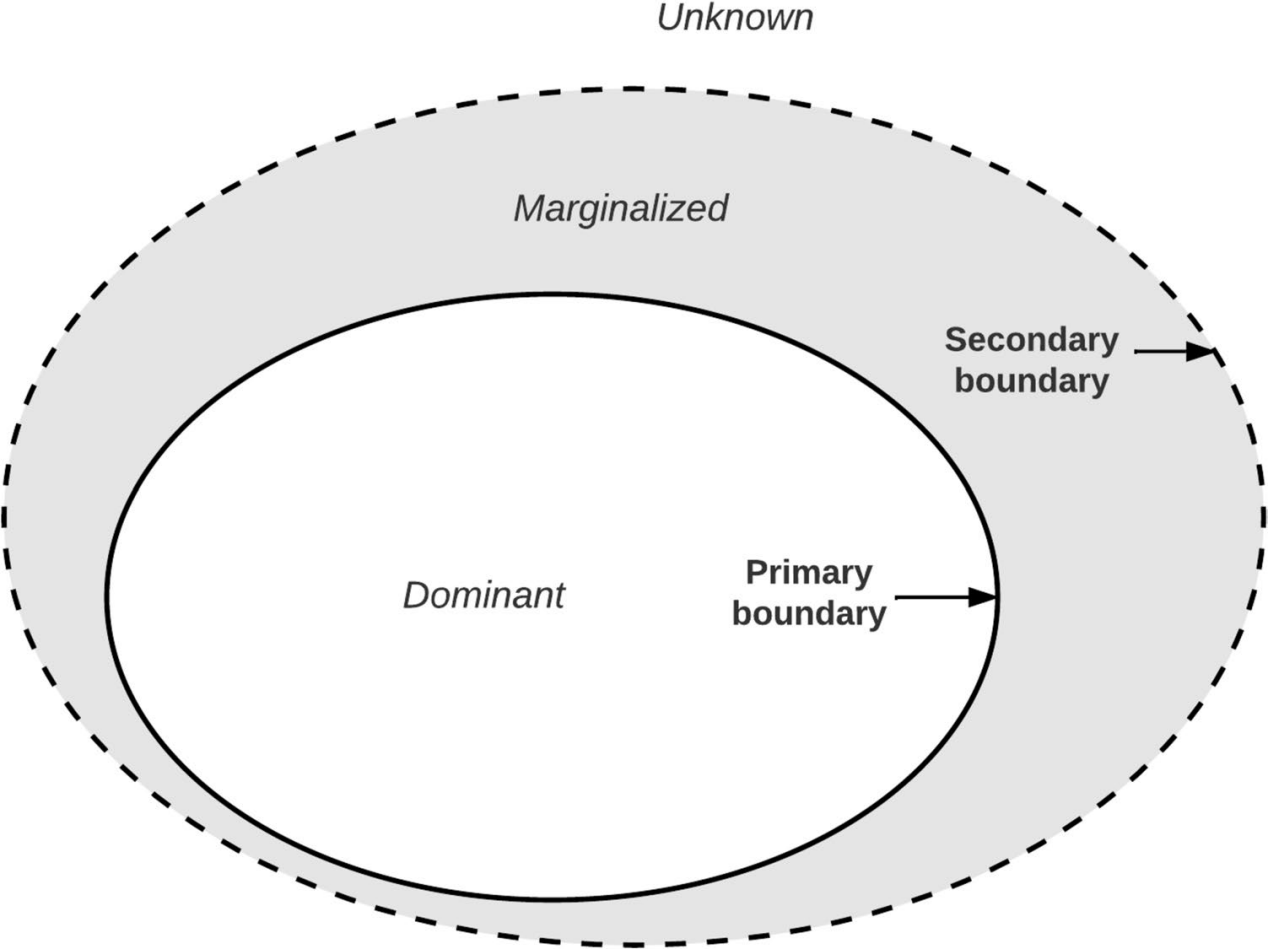
Boundary marginalization (adapted from Midgley 2000)

The imperative for critique in operational research began with its orientation toward intervention (Flood and Jackson 1991; Midgley 2000). This positionality renders the boundary process as a form of intervention because it serves to reinforce or marginalize certain framings, which in turn reinforces or marginalizes certain actor perspectives, interests, and assumptions associated with real-world challenges. Without critique, dominant assumptions remain unquestioned because boundaries are considered objective and absolute, resulting in boundary marginalization as depicted in Fig. 2 (Midgley et al. 1998; Midgley 2000).

Boundary marginalization characterizes the power dynamics between frames derived from different boundary judgments. In boundary marginalization, a primary boundary delineates what is *included* in the analysis, and a secondary boundary encompasses everything that is *known but excluded* (Midgley 2000; Rajagopalan and Midgley 2015). The conceptual region beyond the primary boundary is marginalized, and anything beyond the secondary boundary is unknown. The hardening of boundaries occurs in the absence of critique when specific boundary judgments are stabilized and reinforced by social rituals and stereotypes. According to Midgley (2000), if the primary boundary is privileged, elements in the marginal area can be disparaged and become 'profane'. If the secondary boundary attracts attention and is reinforced, then the marginal elements become the focus of attention and are made ‘sacred'. This dynamic justifies the need to ‘sweep in’ relevant information and perspectives. For example, operational researchers working with Social Service Departments in the UK recruited elderly populations in stakeholder engagement. Doing so was an effort to avoid marginalizing their views, which could disparage their perspective and justify their exclusion from social support (Midgley et al. 1998).

A conceptualisation of ambiguity that directly addresses the political and ethical implications of multiple valid frames is needed. Like systemists, transdisciplinary sustainability researchers are also oriented toward intervention, as they and their research are embedded in the systems and problems they are attempting to understand, motivating efforts to more effectively link knowledge to solutions and action (Kates et al. 2001; van Kerkhoff and Lebel 2006; Miller 2014; Miller et al. 2014; Clark et al. 2016; Caniglia et al. 2017; Preiser et al. 2018). Moreover, the increasingly transformative agenda of sustainability science demands intervention that ‘opens up’ dominant frames (i.e., or ‘sweeps in’ marginalized frames, in the language of CST) to embrace the novel ideas and practices from which transformation emerges (Westley et al. 2011; Moore et al. 2014). Thus, boundary marginalization is a relevant means to characterize the power dynamics between frames that influence transdisciplinary research and its implications for action. For example, a recent review showed that Indigenous and local knowledge is currently neglected in transformation research (Lam et al. 2020). This marginalization is a reflection of historical epistemic injustices that place scientific knowledge holders in positions of power and safe from critique (Cundill et al. 2005; Cote and Nightingale 2012; Gregory et al. 2020). A similar dynamic applies to the privileging of certain perspectives over others in seemingly benign forms of knowledge integration, such as when qualitative social sciences are translated into quantitative natural sciences frameworks or models. In such cases, marginalization is perpetuated through political dynamics that render dominant frames (i.e., within primary boundaries) as neutral and objective and marginalized frames (i.e., within secondary boundaries) as political and subjective (Turnhout 2018; Turnhout et al. 2020). Importantly, these processes of marginalization—and conversely the accrual of power—are not only political but constitute real-world interventions. For example, overriding Indigenous knowledges in natural resource management forces an ontological disruption in the relationship between humans and nature that elevates solutions associated with environmental degradation and social harm. Thus, CST presents an opportunity to link the political nature of efforts to develop more holistic knowledge about complexity (Sarewitz 2010) with broader discussions about how to more effectively link knowledge to action (Matson et al. 2016), and in particular the extent to which the field can generate transformational solution options (Wiek et al. 2012).

## Operationalizing ambiguity in sustainability science

The discussion about the holistic conceptualization of ambiguity in "Conceptualizing ambiguity in sustainability science" points to two recommendations for operationalizing ambiguity in sustainability science. First, "Critique our frames: reflexive boundary critique" suggests the need to *nurture the capacities of transdisciplinary researchers to navigate ambiguity* as a fundamental part of rigorous sustainability science. We offer an operational framework of Reflexive Boundary Critique to help do so, which we adapted from CSH and refined through four case study reflections. Following this discussion, "Broaden our orientation: theoretical incommensurability and discordant pluralism" suggests that for researchers to be able to navigate ambiguity through reflexivity, we need to *broaden our theoretical orientation* to grapple with all three boundary processes associated with ambiguity (i.e., being, knowing, and intervening), allowing for consideration of the potential for and consequences of theoretical incommensurability and discordant pluralism.

### Critique our frames: reflexive boundary critique

Our first recommendation for operationalizing ambiguity is to *nurture the capacities of transdisciplinary researchers to navigate ambiguity* as a fundamental part of the research process. This suggestion emerged from the recognition of the pitfalls of meta-theories and frameworks for directing pluralism in CST (see discussion about how any meta-framework is itself a frame in "Processes of being: observer-dependence"), which directed more attention to the capacities of individual systemists as they navigated through the irreducible ambiguities inherent to theoretical and methodological pluralism in systems practice (Bowers 2019).

#### The need for reflexivity

Reflexivity is cited as a crucial capacity for navigating ambiguity and pluralism in transdisciplinary research (Popa et al. 2015; Moore et al. 2018). The concept of reflexivity has been explored from various perspectives (e.g., Fook 1999; Salzman 2002; Johnson and Duberley 2003; Archer 2016), broadly involving the process of examining how one’s own beliefs, judgments, and practices influence the research. In other words, reflexivity can help translate ambiguity from a slippery phenomenon ‘out there’ to a process that can be embedded in a research process. It may also facilitate more effective links between knowledge and action: conservation scientists have also called for “knowledge systems analysis”, a form of reflexivity that aims to stimulate a better understanding of why knowledge may, or may not, effectively inform decision-making (Miller et al. 2010). Yet, frameworks and tools for nurturing reflexivity in sustainability science are still under development and do not offer expansive guidance to operationalize the three boundary processes (i.e., being, knowing, and intervening) that contribute to ambiguity. For example, the ‘undisciplinary compass’ highlights key capacities required to navigate the *processes of being and knowing* associated with transdisciplinary sustainability science (Haider et al. 2018). ‘Co-productive agility’ addresses *processes of being and intervening* by detailing how a researcher’s role can reinforce or challenge the status quo in service of transformation (Chambers et al. 2022). Additional literature focuses on a researcher’s power and positionality (Williams 2014; Maclean et al. 2022), the ethical dilemmas that arise from methodological pluralism (West and Schill 2022), and the need for decolonization and unlearning for western-trained scientists to be open to the legitimacy of other ways of being or knowing (Stein et al. 2020). Early career researchers increasingly draw from this literature to reflect on how their positionality and philosophical orientation influence research outcomes (e.g., Haider 2017; Macdonald 2019; González García-Mon 2022). However, this type of reflexivity lacks guiding frameworks and is not currently incentivized within traditional structures of academia.

#### An operational framework

We developed Reflexive Boundary Critique (RBC) as a framework that can be embedded into transdisciplinary research processes to operationalize ambiguity through reflexivity. The framework is underpinned by Midgley’s process philosophy ("Processes of knowing: knowledge as a boundary process") and adapted from CST’s original boundary critique. As discussed in "Processes of knowing: knowledge as a boundary process", boundary critique or critical systems heuristics includes a series of questions that encourage reflection upon the way in which a claim depends on its reference system, and this reference system is a product of boundary judgments (Ulrich 1983; Ulrich and Reynolds 2010). Boundary critique is best applied alongside other systems approaches to provide them with legitimacy (Jackson 2019; Nicholas et al. 2019).

The questions that guide RBC are presented in Table 1. The questions move beyond Ulrich’s original framework, which focused on the process of making boundary judgments about the system (*knowing*), to address the *simultaneous and interacting boundary processes associated with being, knowing, and intervening in complex systems*. Questions are asked under these three boundary processes, in addition to the four categories of the original boundary critique: sources of motivation, power, knowledge, and legitimacy. They are asked in both the ‘is’ and ‘ought’ mode, where responses in the ‘ought’ mode clarify the ethical standpoint from which judgments in the ‘is’ mode are evaluated. Differences in response between these two modes, or between individuals, point to unresolved boundary issues and require greater scrutiny.

**Table 1:** Reflexive boundary critique

Process of ambiguity	Boundary judgments			
	Sources of… motivation	… Power	… Knowledge	… Legitimacy
*Being* Boundaries of the researcher’s subjective orientation	What is/ought to be my motivation for pursuing this research? What are/ought to be my conditions for desirable change?	What power to facilitate desirable change is/ought to be in my control? What conditions for desirable change (e.g., resources) are/ought to be under my control? Which are/should not?	What is/ought to be the unique knowledge I bring to the research? What is/ought to be my theory orientation and onto-epistemological lens?	Which knowledge is/ought to be considered salient, valid, and legitimate, including incommensurate frames (according to my onto-epistemological lens)?
	How do these sources of motivation, power, knowledge, and legitimacy influence judgments under ‘knowing’ and ‘intervening’?			
*Knowing* Boundaries of the system	Who does/ought to have a stake in this system? What is/ought to be the purpose of this system? What is/ought to be its measure of desirable change for the system?	Who is/ought to have the power to influence desirable change in the system? What conditions for desirable change (e.g., resources) are/ought to be controlled by the powerful? Which are/should not?	Who is/ought to be providing relevant knowledge in the system? What is/ought to be relevant new knowledge in the system? What is/ought to be the guarantor of desirable change (e.g., consensus, inclusion, etc.)?	Who is/ought to be representing the interests and frames of those affected by but not part of the system? What conditions are/ought to secure the emancipation of those affected by but not part of the system? What process is/ought to be in place to integrate and/or validate across different types of knowledge or frames (e.g., visions of desirable change)?
*Intervening* Impact of the boundary process	Whose stakes and visions of desirable change will be/were reinforced or marginalized by the research?	Whose power will be/was reinforced or marginalized by the research?	Whose and what type of knowledge will be/was reinforced or marginalized by the research?	Whose interests and frames will be/were reinforced or marginalized by the research?

Operationalizing the framework requires embedding these questions before, during, and after a research process as needed, both within the research team and among other participants in co-production processes. Doing so aims to facilitate a deliberative form of self-reflective critique about common sources of selectivity in sustainability science, allowing researchers to learn and evolve their practice according to otherwise under-acknowledged practical and ethical boundary processes. By revisiting questions throughout the research process, changes in response may point to emerging unresolved boundary judgments. Importantly, this framework should be considered a starting point for reflection to be used, adapted, and expanded upon as required. Further, it should not be considered a way of directing and controlling reflexivity (and thus resolving ambiguity), but rather as a navigational tool that helps researchers let go of control as they make sense of, proceed through, and learn from persistent ambiguity and the ethical–political dimensions of research practice (West and Schill 2022; Caniglia et al. 2023).

#### Case study reflection

Four of the co-authors used the questions from RBC to reflect upon their own case study research. These reflections helped refine the framework and demonstrate the type of insights that emerge when using RBC to operationalize ambiguity. Key findings from the case studies are summarized in Table 2.

A. Paradoxes of power: indigenous-led monitoring and evaluation in the Northern Territory, Australia (Simon West).

**Table 2:** Summary of case study reflections under categories of ‘being’, ‘knowing’, and ‘intervening’

Case	Being	Knowing	Intervening
A: Paradoxes of power: Indigenous-led monitoring and evaluation in the Northern Territory, Australia	Original motivation was to use an interpretive approach to contribute to a greater understanding of issues in intercultural collaboration, thereby contributing to greater equity Motivation shifted upon recognition that the approach may not directly benefit Indigenous peoples in the region	Entire research approach shifted to support the implementation of an Indigenous-led project that aimed to build an intercultural M&E system Project adopted a multiple evidence based approach to maintain integrity of Indigenous and Western methodologies in a joint M&E framework (i.e., ‘sweep in’ multiple perspectives)	The project explicitly challenged core assumptions of Western planning frameworks (i.e., the dominant boundaries) by centering Indigenous methodologies (i.e., the marginalized) This paradoxically surfaced tensions as Indigenous methodologies were not made neatly commensurable with Western frameworks
B: Making meaning of “just enough”: Alpine dairy practices in Austria	Purpose was to use a process-relational approach to understand resilience of alpine family dairy farming Original motivation was to elicit practices and knowledge from multiple cases (farms) that could contribute to generalized knowledge for food systems transformation Recognition that ‘being’ multiple farming logics at the same time was not possible, led to a shift in approach (co-production)	Research became co-produced through dialog with one innovative farmer Intention shifted to understanding the meaning of being present, in place, in a moment of time and how this affects the decisions we make for the future	This approach challenged traditional power dynamics between researcher and ‘participant’ By shifting away from a conventional case study research approach, the study not only ‘swept in’ marginalized perspectives but centered them through dialog
C: Legitimate to whom? Climate resilient futures in the Red River Basin	Motivation was to enrich scenarios in water sector with a more expansive set of drivers and perspectives (and to test a particular scenario method to do so) Partnership with influential actors lent legitimacy to the study	Synthesized scientific and local/practitioner knowledge in one scenario model, from a critical realist perspective When ambiguity surfaced, used literature to validate and produce one common set of scenarios	Scenario method broadened the scope of the future to include more diverse drivers and perspectives Issues of representation and integration via a single scenario model marginalized certain perspectives, affecting the legitimacy of outcomes
D: Causality and fisheries collapse in the Baltic Sea	Motivation to reveal different notions of causality and their implications for conceptualizing and addressing environmental challenges Researcher’s particular onto-epistemological commitments helped realize this motivation	Research approach was formulated to make the argument that marginalized understandings of causality (e.g., in terms of intra-action) may imply conceptualizing environmental challenges differently This may point to other, potentially interesting intervention points that may seldom be in focus	The motivation and approach (revealing different understandings of causality and how they point to different intervention points) were formulated to highlight and give room to marginalized boundaries

I arrived in Northern Australia as a White, male, cisgendered visiting researcher from a European institution with a specific positionality, aim, and motivation (*processes of being)*. I was interested in applying theories and methods from interpretive policy analysis (developed within Western scholarly traditions) to descriptively explore tensions between Western scientists and Indigenous peoples working together in the field of Indigenous Land and Sea Management (ILSM). I initially felt that this approach might contribute to a greater understanding of the issues in intercultural collaboration, which could contribute to greater equity for Indigenous peoples in land management partnerships. However, as I embarked on the research, I began to question the ethical and practical value of pursuing such an approach, which might have benefited my agenda as a researcher more than the interests of Indigenous peoples in the region. I gradually found myself shifting into a role of volunteering and helping to fulfill one of the (Indigenous-led) projects I had initially come to descriptively study. This project—the Intercultural Monitoring and Evaluation Project (IMEP)—aimed to bring together Indigenous methodologies and participatory action research to build an intercultural monitoring and evaluation system for an Indigenous ranger group as part of ongoing efforts to develop Indigenous-led approaches to land management in the region (*processes of knowing;* Campion et al. 2023). My motivation had therefore changed from seeking to fulfill my own methodological interests toward contributing to the initiative of the Indigenous rangers and Traditional Owners.

The IMEP project engaged both Indigenous and Western methodologies through a multiple-evidence-based approach, which aimed to retain the integrity of both knowledge systems within a monitoring and evaluation (M&E) framework still recognizable within Western funding and governance systems. This strategic approach of ‘sweeping in’ multiple perspectives was led by senior Indigenous rangers and had already contributed to several tangible beneficial interventions in the region (*processes of intervening*). However, this approach also raised tensions when Indigenous methodologies were positioned in a central role on their terms, without being made neatly commensurable with Western approaches and findings. For example, while the use of Indigenous methodologies arguably enhanced the value and legitimacy of the emerging M&E system in the eyes of Indigenous rangers, local clans and Traditional Owners, it may have reduced the comprehensibility of the system for some non-Indigenous scientists, planners, and policy actors in the broader ILSM governance network. Therefore, by explicitly challenging core assumptions and concepts of Western planning frameworks, and explicitly respecting and working with ambiguity generated by differences between knowledge systems, the risk was that IMEP might paradoxically reinforce or at least fail to address the marginalization of Indigenous interests from dominant processes in ILSM (at least in the short term). This highlights the ethical and political dimensions of strategically adopting and contesting boundary judgments, as well as their unavoidably interventionist character.

B. Making meaning of “just enough”: alpine dairy practices in Austria (Jamila Haider).

This study of alpine farming resilience was rooted in a strong sense of my subjective frame, mainly a motivation to use a process-relational approach to understand what makes family dairy farming in the Austrian Alps resilient (*processes of being*). I took an ethnographic approach focusing on traditional daily practices of cheese-making. This meant ‘being’ in the process of summer-pasture cheese production and focusing on farming as a practice as opposed to the farm as a unit (Darnhofer 2020). Through the fieldwork, I aimed to observe practices and elicit verbal knowledge through interviews across a number of different farms to synthesis characteristics that contribute to farming resilience and that could be further theorized and scaled (*processes of knowing*). The initial research design had as a primary objective to elicit relevant practices and knowledge and to scale-up on-farm knowledge to contribute to more generalized knowledge required for food-systems transformation.

However, through the process of fieldwork, of ‘being’ in the farming processes, I realized it might be possible ‘know’ multiple farming logics, but it was not possible to ‘be’ multiple farming logics at the same time (*processes of being*), and I therefore decided to focus on just one innovative farmer in Gastein valley: Präa Sepp, one of the few remaining farmers in the valley who processes milk in the summer pastures and who manages his farm enterprise to produce “just enough.” The intention of my data collection shifted from eliciting characteristics of resilience, to understanding what “just enough” is, and making meaning of our dialogs. According to Sepp, our engagement was relational, dialogical and he felt that my aim was “to really understand him, on his land, rather than look for answers that I wanted to hear.”

The research shifted to being co-produced with Präa Sepp *(processes of knowing*). Thus, his own reflexivity, and in particular his capacity to embrace novel or marginalized boundaries, is crucial to the research. For example, contrary to all other farmers in the valley, he only milks his cows once a day. This practice emerged from a crisis: a snowstorm forced the cows down the mountains from the summer pastures, and it became difficult to milk them more than once a day. Since then, the cows produce a bit less milk, but it’s “enough” and Sepp has more time for himself, or for other work, and the cows “have a bit more for themselves too”. In changing the traditional milking pattern, he was able to with-hold the larger tradition of milk processing on the summer pasture. But Sepp does not see this as a tradition. In fact, he sees traditions as “unreflective habits.” And in his view, summer cheese making is not a tradition, but rather a practice that enables him to have autonomy over his own time and production: “just enough to have a good life” for himself and his family. In this sense, the framing of summer cheese-making shifts from being a tradition to an act of resistance against the status-quo of increasing production, and arguably a transformative practice for food system transformation. My frame, as a sustainability scientist, aims to intervene in the food system by elevating and emancipating this perspective of the farmer, directly challenging its marginalization under the more dominant frame of mainstream industrialized agriculture, with the aim to contribute to more sustainable food systems (*processes of intervening*). However, at the moment of writing, I am living between the ‘is’ and the ‘ought’ questions of RBC, resulting in unresolved boundary judgments and surfacing ambiguity and calls for reflexivity on my part. My frame has shifted in that my entanglement in the research process has become part of the coproduced research and that rather than my research elevating marginalized perspectives, it has become *our* perspective, which has more pragmatic aims of understanding the meaning of being present, in place, in a moment of time and how this affects the decisions we make for the future.

C. Legitimate to whom? Climate resilient futures in the Red River Basin (Anita Lazurko).

My initial motivation for this study was a concern that the water sector is building ‘resilience’ to climate change according to a narrow vision of the future that may reinforce unsustainable and unjust systems. I was also motivated by the opportunity to test a novel scenario methodology for its capacity to systematically open up the future to more diverse drivers and perspectives than is typical in the water sector, which often privileges empirical and positivist information and thus excludes social drivers of change or interactions across scales (*processes of being*). As a transdisciplinary researcher familiar with SES theory, I aimed to apply my knowledge to the design and implementation of a transdisciplinary scenario modeling process in the Red River Basin (a transboundary basin shared by the US and Canada). Through discussions with case study partners, the study aimed to co-develop exploratory scenarios that characterize future change as emergent from interactions between diverse efforts to build resilience to climate change and a complex, cross-scale SES (Lazurko et al. 2023). I chose critical realism as the philosophical perspective for the scenario process, which allowed me to synthesize scientific and local/practitioner knowledge in one scenario model. This process of synthesis surfaced significant ambiguities in the research context (including potentially incommensurate frames), which I validated with literature and sensitivity analysis to generate integrated findings that were robust across divergent assumptions (*processes of knowing*).

These framing and methodological choices had implications for how the system was characterized and whose interests and perspectives were reinforced or marginalized. My partnership with influential actors in the river basin lent legitimacy to the study and highlighted the interests and knowledge of those who had access to transboundary governance bodies, potentially marginalizing those of others. My choice to use a particular scenario method underpinned by critical realism provided the appropriate grounds to integrate different data sources, effectively broadening the scope of the future to include diverse drivers and perspectives beyond that which is typical in the water sector. However, the inclusion of Indigenous perspectives was isolated to Indigenous governance experts, which I assumed was required to avoid instrumentalizing or coopting the stories of Elders for a western scientific model. This and other issues of representation directly influenced the scenario outcomes, which were perceived as somewhat claustrophobic to, or unrepresentative of, participants who desired more radical transformations from the status quo. Moreover, the choice to validate incommensurate observations with literature and to use scenarios that were ‘robust’ to these divergences helped secure the legitimacy of the final scenarios in the eyes of dominant actors but may have excluded scenarios that may only be considered plausible under marginalized boundaries (*processes of intervening*).

D. Causality and fisheries collapse in the Baltic Sea (Tilman Hertz).

This study (Hertz and Mancilla Garcia 2021) was strongly informed by my subjective frame, namely my motivation and onto-epistemological perspective (*processes of being*). My motivation was twofold: (1) to show how the constitutive and causal dimensions of an analysis intra-act and (2) that there is no one “correct” way for this intra-action to realize. In debates around causation, a difference is often made between the constitutive dimension (what a system is made of) and the causal dimension (causal processes connecting elements of a system). If we consider the act of defining what a system is made of as partly political/ethical (i.e., making some aspects of reality matter at the expense of others) and further acknowledge that causal and constitutive dimensions intra-act then we can assume that particular causal processes are specific to particular constitutive spaces.

I used the case study of cod collapse in the Baltic Sea because of its paradigmatic character and familiarity in social-ecological scholarship. My hope was to contribute to ongoing work that calls for rethinking the concept of causality (Barad 2012; Barad and Gandorfer 2021) beyond its purely efficient dimension and explore the political/ethical aspects of the constitutive-causal intra-action: Why is reality expressed in a particular way? Whose interest does it serve? What does it conceal and silence? Answering these questions might shift research and practice to consider constitutive spaces inherent in often marginalized perspectives and point to novel intervention points for real-world sustainability challenges (*processes of intervening*)*.* The research question and subsequent boundary judgments in the process of knowing were formulated in such a way as to articulate and make the argument.

This approach was empowered/rendered possible by my own onto-epistemological commitments, which enabled a critique (from a political/ethical point of view) of the particular constitutive space of intelligibility that is specific to modernity (Latour 2005). The study argues with many others that it is a fundamental ethical obligation to keep open the possibilities for understanding reality thus attempting to highlight boundaries that are often marginalized (*processes of intervening*). In saying that the possibilities for understanding reality should be kept open, I took a stance in the domain of 'ought'. My criterion for ‘ought’ was not ‘correspondence with reality’ or ‘coherence with an existing body of beliefs’ but was rather inspired by Isabelle Stengers’ notion of ‘relevance’.

E. Summary.

Key findings from the case study reflections under categories of *processes of being, knowing,* and *intervening* are summarized in Table 2. All four case study reflections indicated that the researcher’s motivation and theoretical orientation were influential to the rest of the boundary judgments, and in one case a shift in motivation based on emerging ethical considerations transformed the research approach. Moreover, ambiguity presents unique challenges at different stages of the research process. For example, early research stages involve navigating the uncomfortable space between the “is” and the “ought” modes of critique through tentative boundary judgments around which a researcher can iterate and learn. This differs to later stages, when judgments may become hardened and critical reflection can help researchers remain open to emergent ethical considerations.

### Broaden our orientation: theoretical incommensurability and discordant pluralism

Our second recommendation is that for sustainability science to effectively embrace the ‘elephant in the room’, i.e., ambiguity and all three boundary processes that contribute to it (*being, knowing*, and *intervening*), the field must broaden its theoretical orientation to grapple with the potential for and consequences of *theoretical incommensurability* and *discordant pluralism.* Theoretical and methodological pluralism is considered an important means for sustainability science to address complex sustainability challenges (Jerneck and Olsson 2020; Clark and Harley 2020), and offers the foundation to expose and grapple with ambiguity in sustainability science. Yet, the philosophical stance underpinning such pluralism is often unclear (Cockburn 2022), leaving researchers in ‘conceptual la-la land’ (Haider et al. 2018) and reinforcing the myriad risks and power dynamics associated with uncritical knowledge integration. Of particular relevance is the risk that knowledge integration based on consensus and integration denies the potential for discordant frames, which risks masking ambiguity as these frames are (inadvertently) discarded or rendered invisible (see discussion in "Processes of being: observer-dependence").

As sustainability scientists make efforts to navigate through the diverse theoretical and methodological orientations of the field (Hertz and Schlüter 2015; Preiser et al. 2021), the experience of critical systems theorists grappling with pluralism (i.e., "Processes of being: observer-dependence") offers an important and timely lesson: the search for any organizing meta-theory or framework for pluralism reflects an assumption of theoretical commensurability that risks masking ambiguity. For example, critical realism has been suggested as an appropriate theoretical orientation for knowledge integration in sustainability science (Biggs et al. 2021; Cockburn 2022). Critical realism is thought to allow multiple frames to coexist without contradiction because it differentiates between the real (but unknowable) and observable worlds, thereby accepting that all knowledge is incomplete (Collier 1994; Bhaskar and Hartwig 2016). This view aligns with the partial, provisional, and contingent nature of knowledge under critical systems theory and may help grapple with the *processes of being and knowing* ("Processes of being: observer-dependence" and "Processes of knowing: knowledge as a boundary process") that contribute to ambiguity. However, every theoretical orientation or framework, including critical realism, is still delineated by deeper ontological boundaries that render certain frames visible or invisible. Thus, an assumption that critical realism can be operationalized as a ‘meta-framework’ risks masking ambiguity, in particular the *processes of intervening* ("Processes of intervening: boundary marginalization") that marginalize less dominant frames. For example, while many frameworks in sustainability science may be compatible with critical realism (e.g., the SES perspective), other less mainstream philosophies may not be; for example, philosophies in which epistemology and ontology are entwined (e.g., many Indigenous philosophies, and posthumanism in Western knowledge systems) are not aligned with critical realism’s distinction between real and observable worlds.

A discussion about the potential for and consequences of *theoretical incommensurability* and *discordant pluralism* in sustainability science is required for TD research to address all three boundary processes (i.e., being, knowing, and intervening) that contribute to ambiguity. Without addressing the potential for incommensurate frames through discordant pluralism, disagreement and conflict between the diverse onto-epistemological orientations of actors involved in knowledge production becomes an unacknowledged ‘elephant in the room’. These unacknowledged power dynamics marginalize important perspectives in sustainability science, including the novel ideas and practices from which transformations to sustainability might emerge. Questions should arise in response to these challenges, such as: under what conditions could or should any specific lens or framework (e.g., critical realism, or the SES lens) serve as a meta-theory for integration within sustainability science? How do we handle incommensurate observations? How can we draw from alternative theories to operationalize discordant pluralism?

## Discussion and conclusions

This paper aimed to explore how key concepts and frameworks from CST may be adapted to conceptualize and operationalize ambiguity in sustainability science. "Conceptualizing ambiguity in sustainability science" offers a definition of ambiguity as an *emergent feature of the simultaneous and interacting boundary processes associated with being, knowing, and intervening in complex systems*. Our definition of ambiguity expands on previous understandings of ambiguity by describing it as an emergent and ever-evolving process and explicitly foregrounding its onto-epistemological and ethical dimensions. Second 4 offers two overarching recommendations for operationalizing our conceptualisation of ambiguity: (1) nurturing the reflexive capacities of researchers and (2) broadening the theoretical orientation of sustainability science to consider theoretical incommensurability and discordant pluralism. These two recommendations offer ways for sustainability scientists to embrace, rather than ignore or side-step, this persistent ‘elephant in the room’.

Our first recommendation recognizes lessons from CST: that amid the pitfalls of directing pluralism via meta-frameworks (e.g., that *how* a method is used is as important as its theoretical origins), greater emphasis should be placed on the capacities and orientations of individual researchers navigating across theories and methods. Sustainability scientists are beginning to address such capacities, including the reflexivity required for transdisciplinary researchers to maintain academic rigor despite the lack of disciplinary guardrails. CST offered the starting point for developing the framework of RBC, which offers a series of questions that aim to expose, mediate, and embrace the boundary processes contributing to ambiguity. Further reflection on the use of RBC as an *ex-poste* reflection tool (i.e., as was done with our case studies) indicates its potential value as an a priori guide to nurture reflexivity in research practice. RBC may serve to explicitly surface dilemmas that were encountered subliminally as constant sources of discomfort, allowing researchers to adapt their approach. In particular, the continual shifting between the ‘is’ and ‘ought’ and explicit focus on *processes of intervening* may give shape to emergent ethical and political considerations. For example, RBC may have motivated Lazurko (Case C) to find an additional study partner who could help ‘sweep in’ more diverse perspectives or adjust the methodology to explore marginalized boundaries. Similarly, Haider (Case B) may have been motivated to co-produce the research from the beginning. However, the use of RBC as an a priori guide may not be quite as linear as the case study reflections appear. Rather, it may help researchers remain open to and embrace the ambiguity that persists through the emergent, entangled, and messy nature of a transdisciplinary research process.

Our second recommendation furthers these lessons from CST: that to operationalize all three boundary processes contributing to ambiguity (such as through reflexivity), sustainability science must broaden its theoretical orientation to grapple with theoretical incommensurability and discordant pluralism. This lesson is relevant and timely, as two streams of sustainability scientists emerge. On one hand, some are advocating for an integrated approach that acknowledges complexity but reduces ambiguity through integration within an overarching disciplinary paradigm (e.g., Clark et al. 2016; Clark and Harley 2020), implicitly viewing ambiguity as undesirable and a barrier to practical solutions. In contrast, others are trying to remain open to—and embrace—complexity through transdisciplinarity and pluralism (Cornell et al. 2013; Turnhout et al. 2019; West et al. 2020; Caniglia et al. 2020), viewing ambiguity as intrinsic to interventions that meaningfully address complex sustainability challenges. The former may serve to legitimize sustainability science from dominant disciplinary perspectives, while the latter may focus more on fulfilling its transformative agenda, which demands an emancipatory approach that exposes and elevates often marginalized framings.

Significant future research is required to operationalize ambiguity in the ways we describe. To the first recommendation, i.e., nurturing the reflexive capacities of researchers (e.g., through RBC), sustainability scientists should embed RBC into research and document the insights. This type of reflexivity is rare in peer-reviewed literature, save isolated examples (McGowan et al. 2014). However, doing so may surface theoretical insights regarding how ambiguity influences research outcomes, in addition to practical insights regarding how ambiguity can be operationalized in pursuit of more rigorous sustainability science. Alternatively, sustainability scientists can integrate boundary critique into the empirical aspects of their study. For example, sensitivity analysis is commonly used to evaluate the influence of data uncertainty on research outcomes, but few attempt to evaluate the influence of different boundary judgments, save isolated examples (Van Asselt and Rotmans 2002). Such efforts may offer insights for embedding reflexivity in broader organizational systems, such as for sensemaking within social-ecological-technological systems (Chester et al. 2023). To the second recommendation (i.e., broadening our theoretical orientation), a starting point is a collective dialogue among sustainability scientists about how ambiguity can be addressed (including incommensurability and discordance) while maintaining the solutions-oriented and use-inspired nature of the field. We suspect that such a dialogue may help sustainability science find a balance between calls for urgent and effective solutions and the need to “keep it complex” (Stirling 2010), for example by establishing more plural, conditional, and deliberative ways of translating science to practice.

We acknowledge that while our definition of ambiguity and the recommendations that followed aimed to be expansive, our efforts to be comprehensive were inevitably limited by the same ambiguity we are attempting to define; see Sarewitz (2010). In other words, our conceptualisation of ambiguity and the RBC framework are underpinned by Midgley’s process philosophy because it is compatible with open systems (i.e., complexity) and addresses the contextual, partial, and provisional nature of knowledge. Yet, by applying process philosophy to develop the integrated framework of RBC, we risk contradicting a core tenet of process philosophy by taking a stance regarding which boundary judgments were most relevant (i.e., process philosophy would view any integrated framework as still necessarily limited). Thus, our framework attempts to give just enough shape to ambiguity to facilitate critical reflection while maintaining that any framework (including ours) is necessarily limited and must be adapted to reflect the needs of different contexts of application.

Readers may find numerous additional aspects of this paper to critique. Embracing ambiguity may place a burden on the research process: reflection about theoretical incommensurability and system boundaries may involve significant time and energy. However, this ‘slowing down’ may be part of a bigger-picture shift needed to ensure the ongoing salience and legitimacy of sustainability science. Others may be dissatisfied with the ambiguity that persists through a paper that aims to operationalize it. Yet, to claim to delineate the boundaries of ambiguity definitively and operationalize it objectively would fall into myriad traps that contradict the rationale for embracing ambiguity in the first place. Thus, we attempted to find a balance that gives ambiguity enough shape to nurture reflexivity (i.e., by defining it as a process, always becoming), while holding our own definition and framework as lightly as possible. We hope this paper provokes and inspires discussion and tangible shifts that further expose and embrace this persistent ‘elephant in the room’.

## References

[CR1] Archer MS (2016). Conversations about reflexivity.

[CR2] Audouin M, Preiser R, Nienaber S (2013). Exploring the implications of critical complexity for the study of social-ecological systems. Ecol Soc.

[CR3] Barad K (2012). On touching—the inhuman that therefore I am. Differences.

[CR4] Barad K, Gandorfer D (2021). Political desirings: yearnings for mattering (,) differently. Theory Event.

[CR5] Bateson G (1979). Mind and nature: a necessary unity.

[CR6] Bhaskar R, Hartwig M (2016). Enlightened common sense: the philosophy of critical realism.

[CR7] Biggs R, Preiser R, de Vos A (2021). The Routledge handbook of research methods for social-ecological systems.

[CR8] Bowers TD (2011). Towards a framework for multiparadigm multimethodologies. Syst Res Behav Sci.

[CR9] Bowers TD (2019) Developments in critical systems theory: on paradigms and incommensurability. In: Proceedings of the 58th meeting of ISSS. Washington, D.C., pp 1–16

[CR10] Brand FS, Jax K (2007). Focusing the meaning(s) of resilience: resilience as a descriptive concept and a boundary object. Ecol Soc.

[CR11] Brandt P, Ernst A, Gralla F (2013). A review of transdisciplinary research in sustainability science. Ecol Econ.

[CR12] Brugnach M, Ingram H (2012). Ambiguity: the challenge of knowing and deciding together. Environ Sci Policy.

[CR13] Campion OB, West S, Degnian K (2023). Balpara: a practical approach to working with ontological difference in indigenous land & sea management. Soc Nat Resour.

[CR14] Caniglia G, Schäpke N, Lang DJ (2017). Experiments and evidence in sustainability science: a typology. J Clean Prod.

[CR15] Caniglia G, Luederitz C, von Wirth T (2020). A pluralistic and integrated approach to action-oriented knowledge for sustainability. Nat Sustain.

[CR16] Caniglia G, Freeth R, Luederitz C (2023). Practical wisdom and virtue ethics for knowledge co-production in sustainability science. Nat Sustain.

[CR17] Cash DW, Adger WN, Berkes F (2006). Scale and cross-scale dynamics: governance and information in a multilevel world. Ecol Soc.

[CR18] Chambers JM, Wyborn C, Klenk NL (2022). Co-productive agility and four collaborative pathways to sustainability transformations. Glob Environ Chang.

[CR19] Chester MV, Miller TR, Muñoz-Erickson TA (2023). Sensemaking for entangled urban social, ecological, and technological systems in the Anthropocene. Npj Urban Sustain.

[CR20] Churchman CW (1970). Operations research as a profession. Manag Sci.

[CR21] Cilliers P (1998) Complexity and postmodernism: understanding complex systems. Routledge, London and New York. ISBN 9780415152877

[CR22] Cilliers P (2001). Boundaries, hierarchies, and networks in complex systems. Int J Innov Manag.

[CR23] Cilliers P (2002) Why we cannot know complex things completely. Emerg Complex Organ. 10.1080/15213250.2002.9687736

[CR24] Clark WC, Harley AG (2020). Sustainability science: toward a synthesis. Annu Rev Environ Resour.

[CR25] Clark WC, van Kerkhoff L, Lebel L, Gallopin GC (2016). Crafting usable knowledge for sustainable development. Proc Natl Acad Sci.

[CR26] Cockburn J (2022). Knowledge integration in transdisciplinary sustainability science: tools from applied critical realism. Sustain Dev.

[CR27] Collier A (1994). Critical realism: an introduction to Roy Bhaskar’s philosophy.

[CR28] Cornell S, Berkhout F, Tuinstra W (2013). Opening up knowledge systems for better responses to global environmental change. Environ Sci Policy.

[CR29] Cote M, Nightingale AJ (2012). Resilience thinking meets social theory: situating social change in socio-ecological systems (SES) research. Prog Hum Geogr.

[CR30] Cundill GNR, Fabricius C, Marti N (2005). Foghorns to the future: using knowledge and transdisciplinarity to navigate complex systems. Ecol Soc.

[CR31] Darnhofer I (2020). Farming from a process-relational perspective: making openings for change visible. Sociol Ruralis.

[CR32] Dewulf A, Biesbroek R (2018). Nine lives of uncertainty in decision-making: strategies for dealing with uncertainty in environmental governance. Policy Soc.

[CR33] Dewulf A, Klenk N, Wyborn C, Lemos MC (2020). Usable environmental knowledge from the perspective of decision-making: the logics of consequentiality, appropriateness, and meaningfulness. Curr Opin Environ Sustain.

[CR34] Escobar A (2018). Designs for the pluriverse: radical interdependence, autonomy, and the making of worlds.

[CR35] Fazey I, Schäpke N, Caniglia G (2020). Transforming knowledge systems for life on earth: visions of future systems and how to get there. Energy Res Soc Sci.

[CR36] Flood RL, Jackson MC (1991). Total systems intervention: a practical face to critical systems thinking. Syst Pract.

[CR37] Flood RL, Ulrich W (1990). Testament to conversations on critical systems thinking between two systems practitioners. Syst Pract.

[CR38] Folke C (2016). Resilience (republished). Ecol Soc.

[CR39] Folke C, Carpenter SR, Walker B (2010). Resilience thinking: integrating resilience, adaptability and transformability. Ecol Soc.

[CR40] Fook J (1999). Reflexivity as method. Annu Rev Health Soc Sci.

[CR41] Funtowicz S, Ravetz J (1990). Uncertainty and quality in science for policy.

[CR42] Gao F, Li M, Nakamori Y (2003). Critical systems thinking as a way to manage knowledge. Syst Res Behav Sci.

[CR43] Gieryn TF (1983). Boundary-work and the demarcation of science from non-science: strains and interests in professional ideologies of scientists. Am Sociol Rev.

[CR44] González García-Mon B (2022). Harvesting from land and sea: social relationships, trade networks, and spatial connectivity in changing social-ecological systems.

[CR45] Goodchild M (2021). Relational systems thinking: that’s how change is going to come, from our earth mother. J Awareness-Based Syst Change.

[CR46] Goodman N (1978) Ways of worldmaking. Hackett Publishing Co, Inc., Indianapolis, US. ISBN 9780915144518

[CR47] Gregory WJ (1996). Discordant pluralism: a new strategy for critical systems thinking. Syst Pract.

[CR48] Gregory AJ, Atkins JP, Midgley G, Hodgson AM (2020). Stakeholder identification and engagement in problem structuring interventions. Eur J Oper Res.

[CR49] Gunderson L, Holling CS (2003). Part IV—linking theory to practice. Panarchy: understanding transformations in human and natural systems.

[CR50] Haider LJ (2017). Development and resilience: re-thinking poverty and intervention in biocultural landscapes.

[CR51] Haider LJ, Hentati-Sundberg J, Giusti M (2018). The undisciplinary journey: early-career perspectives in sustainability science. Sustain Sci.

[CR52] Haraway D (1988). Situated knowledges: the science question in feminism and the privilege of partial perspective. Fem Stud.

[CR53] Harding S (1995). “Strong objectivity”: a response to the new objectivity question. Synthese.

[CR54] Helfgott A (2018). Operationalising systemic resilience. Eur J Oper Res.

[CR55] Hertz T, Schlüter M (2015). The SES-Framework as boundary object to address theory orientation in social–ecological system research: the SES-TheOr approach. Ecol Econ.

[CR56] Hertz T, Mancilla Garcia M, Schlüter M (2020). From nouns to verbs: how process ontologies enhance our understanding of social-ecological systems understood as complex adaptive systems. People Nat.

[CR57] Hertz T, Mancilla Garcia M (2021). The Cod and the Cut: intra-active intuitions. Front Sociol.

[CR58] Hill R, Adem Ç, Alangui WV (2020). Working with Indigenous, local and scientific knowledge in assessments of nature and nature’s linkages with people. Curr Opin Environ Sustain.

[CR59] Jackson MC (2019). Critical systems thinking and the management of complexity.

[CR60] Jackson MC, Keys P (1984). Towards a system of systems methodologies. J Oper Res Soc.

[CR61] Jerneck A, Olsson L (2020) Theoretical and methodological pluralism in sustainability science. In: Mino T, Kudo S (eds) Framing in sustainability science: theoretical and practical approaches. pp 17–34. 10.1007/978-981-13-9061-6

[CR62] Johnson P, Duberley J (2003). Reflexivity in management research. J Manag Stud.

[CR63] Juarrero A (1999). Dynamics in action: intentional behavior as a complex system.

[CR64] Kates RW, Clark WC, Corell R (2001). Sustainability science. Sci Compass.

[CR65] Klenk N, Meehan K (2015). Climate change and transdisciplinary science: problematizing the integration imperative. Environ Sci Policy.

[CR66] Klenk NL, Meehan K (2017). Transdisciplinary sustainability research beyond engagement models: toward adventures in relevance. Environ Sci Policy.

[CR67] Kuhn TS (1970). The structure of scientific revolutions.

[CR68] Lam DPM, Hinz E, Lang DJ (2020). Indigenous and local knowledge in sustainability transformations research: a literature review. Ecol Soc.

[CR69] Lang DJ, Wiek A, Bergmann M (2012). Transdisciplinary research in sustainability science: practice, principles, and challenges. Sustain Sci.

[CR70] Latour B (2005). Reassembling the social—an introduction to Actor-Network-Theory.

[CR71] Lazurko A, Schweizer V, Armitage D (2023). Exploring “big picture” scenarios for resilience in social–ecological systems: transdisciplinary cross-impact balances modeling in the Red River Basin. Sustain Sci.

[CR72] Leach M, Scoones I, Stirling A (2007) Pathways to Sustainability: an overview of the STEPS Centre approach. STEPS Approach Pap 19

[CR73] Leach M, Scoones I, Stirling A (2010). Dynamic sustainabilities: technology, environment, social justice.

[CR74] Levin S (1999). Fragile dominion: complexity and the commons.

[CR75] Levin S, Xepapadeas T, Crépin A-S (2013). Social–ecological systems as complex adaptive systems: modeling and policy implications. Environ Dev Econ.

[CR76] Macdonald J (2019) Looking after Country—towards an understanding of Indigenous perspectives on success

[CR77] Maclean K, Woodward E, Jarvis D (2022). Decolonising knowledge co-production: examining the role of positionality and partnerships to support Indigenous-led bush product enterprises in northern Australia. Sustain Sci.

[CR78] Martin DH (2012). Two-eyed seeing: a framework for Indigenous approaches to Indigenous health research. Can J Nurs Res.

[CR79] Matson P, Clark WC, Andersson K (2016). Pursuing sustainability: a guide to the science and practice.

[CR80] Matthews D, van Gigch JP, McIntyre-Mills J (2006). Pragmatism meets systems thinking: the legacy of C. West Churchman. Volume 1: rescuing the enlightenment from itself.

[CR81] McGowan KA, Westley F, Fraser EDG (2014). The research journey: travels across the idiomatic and axiomatic toward a better understanding of complexity. Ecol Soc.

[CR82] Midgley G (1989). Critical systems and the problem of pluralism. Cybern Syst.

[CR83] Midgley G (1992). Pluralism and the legitimation of systems science. Syst Pract.

[CR84] Midgley G (2000). Systemic intervention: philosophy, methodology, and practice.

[CR85] Midgley G (2011). Theoretical pluralism in systemic action research. Syst Pract Action Res.

[CR86] Midgley G, Munlo I, Brown M (1998). The theory and practice of boundary critique: developing housing services for older people. J Oper Res Soc.

[CR87] Miller TR (2013). Constructing sustainability science: emerging perspectives and research trajectories. Sustain Sci.

[CR88] Miller T (2014). Reconstructing sustainability science: knowledge and action for a sustainable future.

[CR89] Miller TR, Baird TD, Littlefield CM (2008). Epistemological pluralism: reorganizing interdisciplinary research. Ecol Soc.

[CR90] Miller C, Muñoz-Erickson TA, Monfreda C (2010) Knowledge systems analysis: a report for the advancing conservation in a Social Context Project. Consortium for Science, Policy and Outcomes, Arizona State University, Tempe, AZ. https://cspo.org/library/knowledge-systems-analysis-a-report-for-the-advancing-conservation-in-a-social-context-project/

[CR91] Miller TR, Wiek A, Sarewitz D (2014). The future of sustainability science: a solutions-oriented research agenda. Sustain Sci.

[CR92] Moore M-L, Tjornbo O, Enfors E (2014). Studying the complexity of change: toward an analytical framework for understanding deliberate social-ecological transformations. Ecol Soc.

[CR93] Moore M-L, Olsson P, Nilsson W (2018). Navigating emergence and system reflexivity as key transformative capacities: experiences from a Global Fellowship program. Ecol Soc.

[CR94] Morin E (2008). Restricted complexity, general complexity. Worldviews Sci US Philos Complex.

[CR95] Nicholas G, Foote J, Kainz K (2019). Towards a heart and soul for co-creative research practice: a systemic approach. Evid Policy.

[CR96] Ocholla D (2007). Marginalized knowledge: an agenda for Indigenous knowledge development and integration with other forms of knowledge. Int Rev Inf Ethics.

[CR97] Patterson J, Schulz K, Vervoort J (2017). Exploring the governance and politics of transformations towards sustainability. Environ Innov Soc Transit.

[CR98] Peçanha Enqvist J, West S, Masterson VA (2018). Stewardship as a boundary object for sustainability research: linking care, knowledge and agency. Landsc Urban Plan.

[CR99] Popa F, Guillermin M, Dedeurwaerdere T (2015). A pragmatist approach to transdisciplinarity in sustainability research: from complex systems theory to reflexive science. Futures.

[CR100] Preiser R, Cilliers P (2010) Unpacking the ethics of complexity: concluding reflections. In: Cilliers P, Preiser R (eds) Complexity, Difference, and Identity. Springer Netherlands, Dordrecht, pp 265–287

[CR101] Preiser R, Biggs R, De Vos A, Folke C (2018). Social-ecological systems as complex adaptive systems: organizing principles for advancing research methods and approaches. Ecol Soc.

[CR102] Preiser R, Schluter M, Biggs R, Biggs R, de Vos A, Preiser R, Clements H, Maciejewski KMS (2021). Complexity-based social-ecological systems research: philosophical foundations and practical implications. The Routledge.

[CR103] Prigogine I, Stengers (1984). Order out of chaos: man’s new dialogue with nature.

[CR104] Rajagopalan R, Midgley G (2015). Knowing differently in systemic intervention. Syst Res Behav Sci.

[CR105] Rathwell KJ, Armitage D, Berkes F (2015). Bridging knowledge systems to enhance governance of the environmental commons: a typology of settings. Int J Commons.

[CR106] Reyers B, Folke C, Moore ML (2018). Social-ecological systems insights for navigating the dynamics of the anthropocene. Annu Rev Environ Resour.

[CR107] Rosen R (1991). Life itself: a comprehensive enquiry into the nature, origin, and fabrication of life.

[CR108] Rosendahl J, Zanella MA, Rist S, Weigelt J (2015). Scientists’ situated knowledge: strong objectivity in transdisciplinarity. Futures.

[CR109] Rutting L, Vervoort J, Mees H, Driessen P (2022). Strengthening foresight for governance of social-ecological systems: an interdisciplinary perspective. Futures.

[CR110] Salzman PC (2002). On reflexivity. Am Anthropol.

[CR111] Sarewitz D, Frodeman R, Thompson Klein J, Mitcham C, Holbrook J (2010). Against holism. The Oxford handbook of interdisciplinarity.

[CR112] Schatzki TR (2002). The site of the social: a philosophical account of the constitution of social life and change.

[CR113] Staffa RK, Riechers M, Martín-López B (2022). A feminist ethos for caring knowledge production in transdisciplinary sustainability science. Sustain Sci.

[CR114] Stein S, Andreotti V, Suša R (2020). Gesturing towards decolonial futures. Nord J Comp Int Educ.

[CR115] Stirling A (2010). Keep it complex. Nature.

[CR116] Stirling A (2014) Emancipating transformations: from controlling “the transition” to culturing plural radical progress. STEPS Working Paper 64, Brighton: STEPS Centre. https://steps-centre.org/wp-content/uploads/Transformations.pdf

[CR117] Strang V (2009). Integrating the social and natural sciences in environmental research: a discussion paper. Environ Dev Sustain.

[CR118] Tengö M, Brondizio ES, Elmqvist T (2014). Connecting diverse knowledge systems for enhanced ecosystem governance: the multiple evidence base approach. Ambio.

[CR119] Turnhout E (2018). The politics of environmental knowledge. Conserv Soc.

[CR120] Turnhout E, Tuinstra W, Halffman W (2019) Interdisciplinarity and the challenge of knowledge integration. In: Environmental expertise: connecting science, policy, and society. pp 152–164. Cambridge University Press, Cambridge. 10.1017/9781316162514.013

[CR121] Turnhout E, Tuinstra W, Halfmann W (2019). Environmental expertise: connecting science, policy and society.

[CR122] Turnhout E, Metze T, Wyborn C (2020). The politics of co-production: participation, power, and transformation. Curr Opin Environ Sustain.

[CR123] Ulrich W (1983). Critical heuristics of social planning: a new approach to practical philosophy.

[CR124] Ulrich W (2003). Beyond methodology choice: critical systems thinking as critically systemic discourse. J Oper Res Soc.

[CR125] Ulrich W, Reynolds M (2010). Critical systems heuristics. Systems approaches to management change: a practical guide.

[CR126] United Nations (2015) Transforming our world: the 2030 agenda for sustainable development

[CR127] Van Asselt MBA, Rotmans J (2002). Uncertainty in integrated assessment modelling. From positivism to pluralism. Clim Change.

[CR128] van Kerkhoff L, Lebel L (2006). Linking knowledge and action for sustainable development. Annu Rev Environ Resour.

[CR129] Vervoort JM, Bendor R, Kelliher A (2015). Scenarios and the art of worldmaking. Futures.

[CR130] Walker WE, Harremoës P, Rotmans J (2003). Defining uncertainty: a conceptual basis for uncertainty management in model-based decision support. Integr Assess.

[CR131] West S, Schill C (2022). Negotiating the ethical-political dimensions of research methods: a key competency in mixed methods, inter- and transdisciplinary, and co-production research. Humanit Soc Sci Commun.

[CR132] West S, Haider J, Sinare H, Karpouzoglou T (2014) Beyond divides: prospects for synergy between resilience and pathways approaches to sustainability. STEPS Working Paper 65, Brighton: STEPS Centre. https://steps-centre.org/wp-content/uploads/Resilience-and-Pathways.pdf

[CR133] West S, Haider LJ, Stålhammar S, Woroniecki S (2020). A relational turn for sustainability science? Relational thinking, leverage points and transformations. Ecosyst People.

[CR134] Westley F, Olsson P, Folke C et al (2011) Tipping toward sustainability: emerging pathways of transformation. AMBIO 40:762–780. 10.1007/s13280-011-0186-9 10.1007/s13280-011-0186-9 PMC3357751 22338714

[CR135] Whitehead AN (1978). Process and reality, David Ray.

[CR136] Wiek A, Ness B, Schweizer-Ries P (2012). From complex systems analysis to transformational change: a comparative appraisal of sustainability science projects. Sustain Sci.

[CR137] Williams DR (2014). Making sense of “place”: reflections on pluralism and positionality in place research. Landsc Urban Plan.

